# Eculizumab as rescue therapy in severe resistant lupus nephritis

**DOI:** 10.1093/rheumatology/kev307

**Published:** 2015-08-27

**Authors:** Matthew C. Pickering, Mediola Ismajli, Marie B. Condon, Nicola McKenna, Angela E. Hall, Liz Lightstone, H. Terence Cook, Thomas D. Cairns

**Affiliations:** ^1^Imperial College NHS Healthcare Trust Lupus Centre and; ^2^Clinical Immunology, Imperial College Healthcare NHS Trust, London, UK

Rheumatology key message

- Lupus nephritis may respond to complement C5 inhibition.

**Fig. 1:**
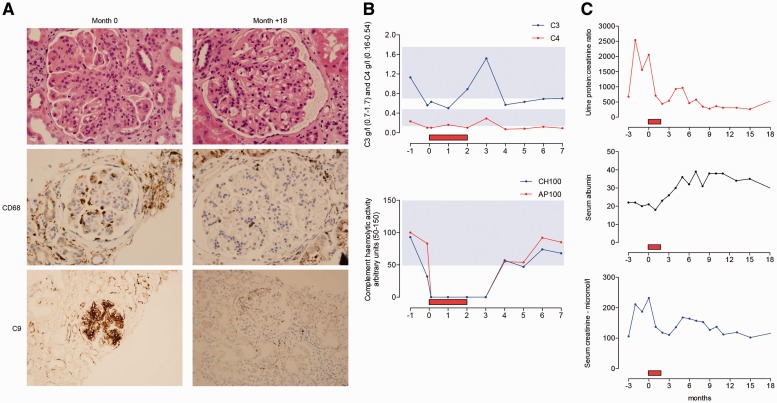
Renal histology, complement profile and renal function during eculizumab treatment **(A)** Glomerular images from renal biopsies immediately before and 18 months after eculizumab. Immunoperoxidase staining for CD68 (middle panels) and complement C9 (lower panels) using PGM1 and NCL-CCC9 antibodies, respectively. Upper and middle panels are ×40 original magnification. Lower panel is ×20 original magnification. (**B**) Serum C3, C4, total (CH100) and alternative pathway (AP100) haemolytic activity are shown 1 month prior to and for 7 months following eculizumab. Shaded areas denote normal ranges. (**C**) Serum creatinine (μmol/l), serum albumin (g/l) and urine protein:creatinine ratio (mg/mmol) are shown 3 months prior to and for 18 months following eculizumab. Red box denotes the 2-month eculizumab treatment period.

Sir, LN is a serious complication of SLE that can be refractory to existing immunosuppressive treatment. We present the clinical course of a patient with refractory LN who was treated with eculizumab, a monoclonal anti-complement C5 antibody therapy [1]. A Caucasian female with SLE first presented with immune thrombocytopaenic purpura at the age of 14 years. Three years later she developed polyarthralgia, oral ulcerations, RP, alopecia and pleurisy. Anti-nuclear and anti-dsDNA antibodies were positive; C3 and C4 levels were reduced; antibodies to extractable nuclear antigens and cardiolipin and LA were all negative. The urine protein:creatinine ratio was 14 (normal <20 mg/mmol), serum creatinine 75 μmol/l (normal range 60–110) and albumin 36 g/l (normal range 33–47). Disease control was achieved with prednisolone and HCQ, but 2 years later she developed proteinuria (protein:creatinine ratio 1123, albumin 27 g/l, creatinine 110 μmol/l) and renal biopsy showed florid diffuse proliferative GN, International Society of Nephrology/Renal Pathology Society class IV-G (A) LN, with active lesions in 10/10 glomeruli and crescents in 5/10 glomeruli. She was treated with six i.v. 500-mg pulses of CYC, rituximab infusions (two 1-g infusions 14 days apart) and then maintenance therapy with MMF. At 12 months she had partial improvement (protein:creatinine ratio 240, albumin 29 g/l, creatinine 92 μmol/l), but at 15 months renal function deteriorated (protein:creatinine ratio 761, albumin 26 g/l, creatinine 93 μmol/l) and repeat renal biopsy showed ongoing active class IV LN. A further cycle of CYC and rituximab was administered followed by tacrolimus monotherapy. Over the course of the next 22 months, impaired renal function persisted. A third renal biopsy, taken due to a further deterioration in renal function (protein:creatinine ratio 1082, albumin 27 g/l, creatinine 108 μmol/l), revealed diffuse proliferative LN class IV-G (A/C). MMF was added, but renal function continued to decline (protein:creatinine ratio 2540, albumin 22 g/l, creatinine 211 μmol/l) and a fourth renal biopsy, 4 years after the first renal biopsy, showed class IV-G (A/C) LN with active lesions in 20 out of 36 glomeruli, chronic lesions in 7 out of 36 glomeruli, acute tubular damage with foci of lymphocytic tubulitis and marked chronic inflammatory interstitial infiltrate. There was granular mesangial and capillary wall staining for C9 consistent with activation of the complement terminal pathway (Fig. 1A shows a representative glomerulus with marked lobulation, mesangial proliferation, capillary wall thickening and prominent endocapillary hypercellularity with occlusion of capillary lumens. CD68 staining shows several macrophages in the glomerulus). In view of her previous poor response to CYC, rituximab, MMF and tacrolimus, together with biopsy-proven evidence of ongoing glomerular inflammation and complement C5 activation, it was decided to administer a single i.v. methylprednisolone 500-mg infusion, together with eculizumab. She received four 1200-mg weekly eculizumab infusions followed by two 1200-mg fortnightly infusions. Complement haemolytic activity remained undetectable following the first infusion and for 1 month after the sixth infusion (Fig. 1B). Antibiotic prophylaxis was instigated throughout the eculizumab treatment period. During and following eculizumab treatment, there was a sustained and rapid improvement in her renal function (Fig. 1C). Following eculizumab therapy she was recommenced on MMF. Renal biopsy, 18 months following eculizumab treatment showed mesangial proliferation, capillary wall thickening and segmental glomerulosclerosis, but no endocapillary hypercellularity or necrosis, and C9 staining was almost undetectable. Consistent with this, staining for CD68, a macrophage marker, was markedly reduced (Fig. 1A).

The current management of LN includes corticosteroids and cytotoxic agents such as CYC, MMF and AZA, but there is increasing use of biologic therapy, most commonly rituximab [2]. Complement activation is common in lupus tissue injury [3], and blockade of complement C5 has been shown to ameliorate murine SLE [4]. Eculizumab is a recombinant fully humanized IgG2/IgG4 monoclonal antibody that binds to complement component C5 and prevents the cleavage of C5 and therefore the formation of the anaphylatoxin C5a and the membrane attack complex (C5b-9) [1]. Eculizumab has efficacy in the prevention of red cell lysis in paroxysmal nocturnal haemoglobinuria [1] and in the treatment of atypical haemolytic uraemic syndrome [5]. There have been reports of its efficacy in C3 glomerulopathy [6], but only one report of efficacy in LN [7]. However, in this case, the renal pathology was thrombotic microangiopathy (TMA), not GN. Efficacy has also been reported in TMA in the setting of IgA nephropathy [8]. To our knowledge, there are no reports of the use of eculizumab to treat non-TMA glomerular lesions in SLE. Our case indicates that, in the right setting, eculizumab may have therapeutic utility in LN. In a complex condition like LN, patient stratification will be critical for rigorously evaluating the potential benefits of eculizumab. Two criteria, which are not mutually exclusive, would include TMA and reversible GN in which there is evidence of C5 activation within the kidney.
